# Estimating Patient Satisfaction Through a Language Processing Model: Model Development and Evaluation

**DOI:** 10.2196/48534

**Published:** 2023-09-14

**Authors:** Shinichi Matsuda, Takumi Ohtomo, Masaru Okuyama, Hiraku Miyake, Kotonari Aoki

**Affiliations:** 1 Drug Safety Division Chugai Pharmaceutical Co Ltd Tokyo Japan; 2 Initiative Inc Tokyo Japan

**Keywords:** breast cancer, internet, machine learning, natural language processing, natural language-processing model, neural network, NLP, patient satisfaction, textual data

## Abstract

**Background:**

Measuring patient satisfaction is a crucial aspect of medical care. Advanced natural language processing (NLP) techniques enable the extraction and analysis of high-level insights from textual data; nonetheless, data obtained from patients are often limited.

**Objective:**

This study aimed to create a model that quantifies patient satisfaction based on diverse patient-written textual data.

**Methods:**

We constructed a neural network–based NLP model for this cross-sectional study using the textual content from disease blogs written in Japanese on the Internet between 1994 and 2020. We extracted approximately 20 million sentences from 56,357 patient-authored disease blogs and constructed a model to predict the patient satisfaction index (PSI) using a regression approach. After evaluating the model’s effectiveness, PSI was predicted before and after cancer notification to examine the emotional impact of cancer diagnoses on 48 patients with breast cancer.

**Results:**

We assessed the correlation between the predicted and actual PSI values, labeled by humans, using the test set of 169 sentences. The model successfully quantified patient satisfaction by detecting nuances in sentences with excellent effectiveness (Spearman correlation coefficient [ρ]=0.832; root-mean-squared error [RMSE]=0.166; *P*<.001). Furthermore, the PSI was significantly lower in the cancer notification period than in the preceding control period (−0.057 and −0.012, respectively; 2-tailed t_47_=5.392, *P*<.001), indicating that the model quantifies the psychological and emotional changes associated with the cancer diagnosis notification.

**Conclusions:**

Our model demonstrates the ability to quantify patient dissatisfaction and identify significant emotional changes during the disease course. This approach may also help detect issues in routine medical practice.

## Introduction

In any service industry, the goal is to identify and respond to customer needs [1]. In health care, customers are patients, and services must be provided based on whether patients are satisfied with the diagnoses, treatment, and care that they receive. Additionally, in the medical field, patient satisfaction can be considered in the context of pharmacovigilance (PV). PV is related to monitoring and minimizing the risks of unfavorable events associated with medications, such as adverse drug reactions, and optimizing the benefit-risk profile throughout the drug life cycle [2]. Recently, in the wake of technological data innovations, some traditional PV activities have faced challenges in improving efficiency and quality [3]. To improve PV, it is essential to consider recruiting patients who have first-hand experience of treatments, but this remains insufficient [4]. Traditional measures, such as spontaneous reporting and electronic health care databases, do not contain information related to patients’ emotions; previous research in this area has used questionnaires regarding quality of life (QOL) or patient-reported outcomes [5]. Furthermore, obtaining sufficient and generalizable knowledge is often challenging owing to small sample sizes and limited patient diversity [6].

Insights from patient-derived resources can help improve PV guidelines to better meet patient needs. However, encouraging patient involvement is associated with challenges in obtaining feedback from different patients. Recent PV studies considered data from social networking services (SNSs) [7-9], but research has shown that the benefits of using Twitter and Facebook as patient-derived resources for PV are few [10]. This may be because SNSs contain a significant amount of information unrelated to treatment, making it difficult to extract the necessary data, as the primary scope of these SNSs is not related to the provision of medical care.

Data resources that are primarily focused on collecting patient experiences are rare. An example of a successful method of collecting such information is the web-based patient community operating in the United States, PatientsLikeMe, which has conducted both prospective data collection and evaluation of treatments from the perspective of patients [11]. One conventional method of collecting data regarding patient feedback is through surveys that include questions on QOL. Surveys conducted by PatientsLikeMe use multiple-choice questionnaires to ask patients about their disease complaints [12]. However, as most QOL questions are developed from the perspective of the health care professional, they tend to focus only on the intentions of those asking the questions. A recent study showed that patients with COVID-19 tended to describe broad aspects of care that mattered to them in the comment field, regardless of the focus of the survey question [13]. This highlights the importance of patient-written narratives, as essential opinions of patients may be overlooked if only prespecified questionnaires are used. Another key challenge is obtaining data that reflect the opinions of a wide range of patients receiving various treatments.

Regarding such data, some patients in Japan who had adopted the custom of writing *tōbyōki*, which are diaries about their longitudinal experience with diseases [14], began posting their *tōbyōki* as blogs in the mid-1990s. These *tōbyōki* blogs, in combination with natural language processing (NLP) [15], facilitate a qualitative understanding of treatment experiences and feelings [16,17]. Nonetheless, a qualitative description alone is not sufficient to make decisions that could improve patient care, and effective methods for visualizing patient anxieties and frustrations are needed.

Based on recent research trends, we believe the following 3 research issues should be addressed:

1. Critical information is missed when only computers are used to classify results based on a preset group of options. Thus, the contents need to be reviewed manually. This takes considerable time before starting a discussion for action.
2. Analyzing qualitative data quantitatively to investigate the significance of differences is challenging. For example, sentiment analysis programs can identify a particular sentiment category as “negative” for a specific group of texts based on machine learning; however, this classification alone does not indicate the intensity of that negativity. Although some studies have quantitatively shown the degree of sentiment, the validity of such estimations in the health care domain has been limited [18]. Hence, manual review by humans is required to prioritize issues for action. Thus, a system that can quantitatively assess the impact of qualitative content in a timely fashion is needed.
3. When using supervised machine learning to classify groups according to patients’ comments, researchers must specify the classification groups beforehand. If a patient has a complaint that does not fit into the prespecified classification groups, it is challenging to capture the complaint using previous classification methods. Thus, we believe that these types of classification approaches have led to the loss of a significant percentage of social media data, which is unfortunate because patients’ comments on social media can yield novel, unanticipated complaints given that they do not have the same restrictions that they would have with structured questionnaires [19].

We believe that the quantitative assessment of patient satisfaction using data from the narratives of diverse patient populations is of significant value; however, no such efforts have been reported. If an efficient tool to obtain patient feedback from written texts could be developed, it could be widely applied to improve health care services. In this study, we aimed to create an NLP model that quantifies patient satisfaction based on diverse patient-written textual data.

## Methods

### Description and Processing of Data

We have collected anonymous, publicly available data from *tōbyōki* blogs written in Japanese from the internet [20]. Individual *tōbyōki* blogs were manually tagged by disease based on the blog title or introduction page. Between 1994 and 2020, the most frequently reported disease on *tōbyōki* blogs was breast cancer (n=6669 blog entries), followed by depression (n=3295), cervical cancer (n=1231), and rheumatoid arthritis (n=1144). We focused on breast cancer because it has the largest number of entries and is the most common cancer in women worldwide [21]. Additionally, as the recurrence of breast cancer exerts a severe psychological distress on patients [22], there is a substantial need to evaluate patient satisfaction during treatment.

### Overall Flow of Model Construction and Evaluation

A systematic review revealed no unified definition of patient satisfaction [23]. It is assumed that patient satisfaction is the result of a combination of treatment efficacy, quality of care, and QOL. Here, the patient satisfaction index (PSI) was defined as a numerical value ranging from −1.0 to 1.0, representing the most dissatisfied and satisfied states, respectively. We applied NLP and machine learning (ML) to develop a quantitative method for evaluating PSIs using textual information. Currently, most approaches to text analysis are based on sentiment analysis, which uses a simple sum of each word’s emotional score [24]. However, this approach limits the ability to capture emotional subtleties. Another critical issue when dealing with words is how to consider the context of each sentence. A previous study reported that the performance of ML models for textual data is inadequate in the absence of contextual considerations [25]. To address these issues, we applied a neural network-based model known as bidirectional encoder representation from transformers (BERT) [26]. It uses word embedding, which helps capture sentence nuance more naturally than sentiment analysis [24]. In addition, BERT provides improved performance because it incorporates the context before and after the words to capture nuances better than other models.

**Figure 1:**
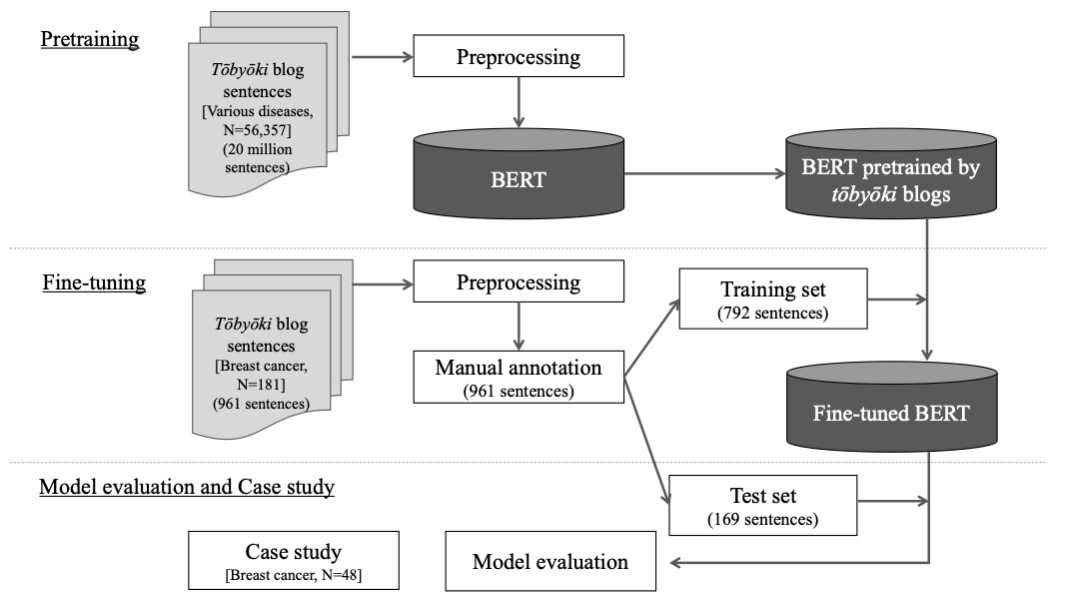
Flow diagram of model construction. The overall flow of model construction consists of a pretraining phase, fine-tuning phase, and model evaluation phase. During pretraining, the bidirectional encoder representation from transformers (BERT) model obtains the spread of words, a mechanism also known as word embedding, from a large data set (in this case, tōbyōki blogs). Next, fine-tuning, a supervised learning process, replaces the output layer of the model for a specific task (in this case, predicting patient satisfaction index [PSI] from the texts).

We attempted to predict PSIs using a regression approach. The overall flow of the model construction is shown in Figure 1, and the data flowchart is shown in Figure S1 in Multimedia Appendix 1. For the training process, we collected 20 million *tōbyōki* blog sentences extracted from disease blogs written by 56,357 patients concerning 1402 distinct diseases, and then the preprocessing was performed to remove the extraneous information (Table S1 in Multimedia Appendix 1) [27]. In general, pretraining requires a vast amount of data and tends to be highly computational and time-consuming. To complete this process efficiently, we implemented transfer learning using the publicly available Japanese version of BERT [28]. We then prepared the training data for fine-tuning, which enabled BERT to predict PSIs. Specifically, we prepared 961 sentences extracted randomly from *tōbyōki* blog entries written by 181 patients with breast cancer. Next, 3 reviewers (SM, TO, and HM) independently reviewed each sentence and provided a numerical value for the PSI as the actual label (PSI labeling guidelines are presented in Table S2 in Multimedia Appendix 1; statistics for annotation results are presented in Table S3 in Multimedia Appendix 1); the mean value of the reviewers’ PSI labels was treated as the actual PSI for each sentence to ensure the model’s robustness by avoiding possible effects of differences between the 3 labels. Subsequently, we randomly divided these sentence data into a training data set (792 sentences) and an unseen test data set (169 sentences) to build the model and evaluate its effectiveness (the characteristics of the data for fine-tuning are presented in Table S4 in Multimedia Appendix 1).

Using the test data set, we conducted a sentiment analysis based on the Japanese sentiment polarity dictionary [29]. Researchers at Tohoku University, a national university in Japan, created this dictionary by collecting nouns and declinable words in Japanese and manually adding polarity information ranging from −1.00 (negative) to 1.00 (positive) [30]. Next, using the same test set, we evaluated the BERT model’s performance based on the correlation between the predicted and actual PSI.

### Change in PSI After Cancer Notification

To confirm the reliability of the prepared BERT model in evaluating the PSI for actual cases, we conducted an evaluation by predicting the PSI before and after cancer notification. The “cancer notification period” was defined as the time when any blog entry containing the word “cancer notification (in Japanese)” was posted; each of these entries was manually confirmed by a reviewer. In contrast, the “control period” was defined as the time covered by 10 entries posted before the 120 days preceding the cancer notification period (Figure S2 in Multimedia Appendix 1). We selected 48 blogs with both a blog entry for the cancer notification period and the preceding control period and computed the PSI in each period; the detailed procedure is presented in Table S5 in Multimedia Appendix 1.

### Statistical Analyses

To evaluate the model’s capacity, the Spearman correlation coefficient (ρ) and the root-mean-squared error (RMSE) were used. A paired, 2-tailed *t* test was conducted to compare the PSIs between the cancer notification and control periods. To compare BERT models with and without pretraining, we used a statistical comparison test for 2 correlations [31]. All statistical analyses were performed using R (version 3.6.2; The R Foundation for Statistical Computing), with *P*<.05 being considered statistically significant.

### Ethical Considerations

We collected anonymous, publicly available data from web-based sources. Per the copyright law issued by the Japanese Agency for Cultural Affairs (Article 47-7), we adhered to regulations allowing the reproduction and adaptation of copyrighted works onto recording media for information analysis within specified limits [32]. To ensure the privacy of individuals, the breast cancer blog data used in this study were meticulously reviewed. They were visually confirmed to be free of personally identifiable information such as the blog authors’ names, handle names, and dates of birth. This study did not necessitate institutional review board approval, aligning with the Ethical Guidelines for Life Sciences and Medical Research Involving Human Subjects [33].

## Results

### Evaluation of the BERT Model

As part of the dictionary-based approach, we conducted sentiment analysis. The correlation between the actual PSI labeled by humans and the predicted sentiment score was very low (ρ=0.127; RMSE=0.563; *P*=.10; Figure S3 in Multimedia Appendix 1). This result shows that sentiment analysis based on the widely used Japanese sentiment polarity dictionary is not appropriate for predicting the PSI.

**Figure 2:**
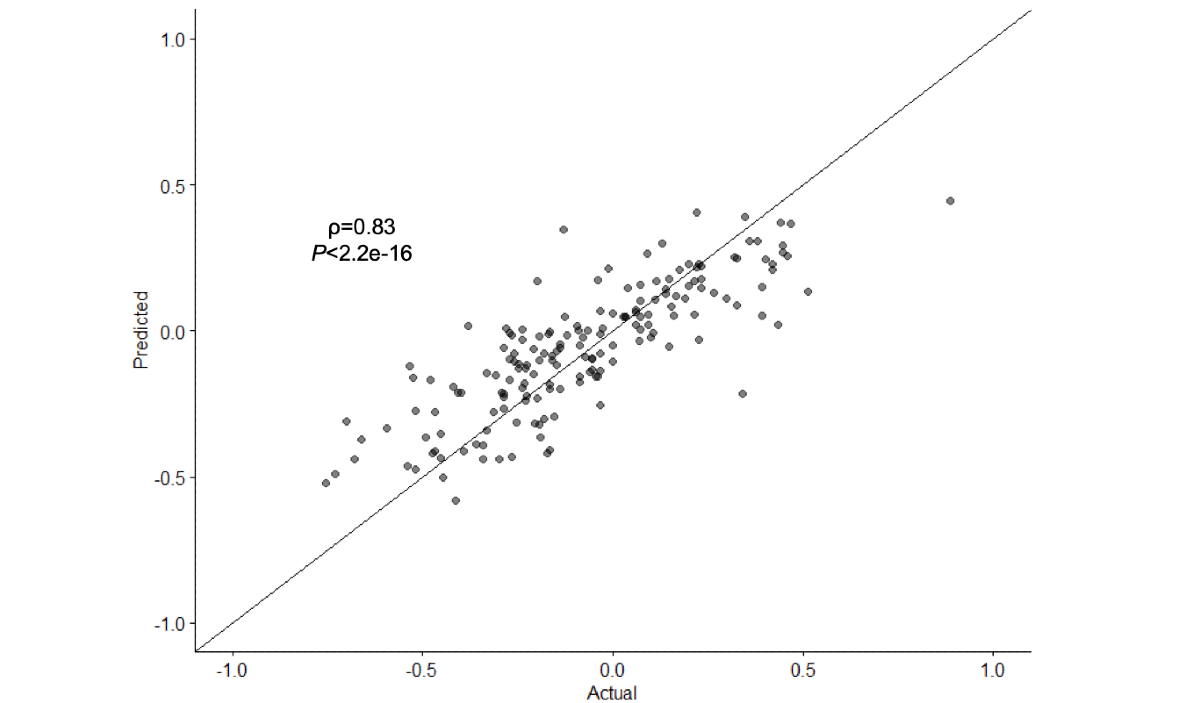
Correlation between the predicted and actual patient satisfaction index (PSI). Predicted versus actual PSI using the test set.

Using the same test set, we evaluated the correlation between the predicted and actual PSI. The BERT model achieved excellent effectiveness as an NLP model for predicting PSIs (ρ=0.832; RMSE=0.166; *P*<.001; Figure 2). PSI prediction using the BERT model without pretraining was less reliable than that using the model with pretraining (ρ=0.554; RMSE=0.250; *P*<.001; Figure S4 in Multimedia Appendix 1). Furthermore, a statistical comparison test for 2 correlations demonstrated that they were significantly different (*P*<.001). This reaffirms that the model’s performance is the highest in the BERT model with pretraining.

We reviewed the sentences with the highest and lowest PSIs (Table 1) and found that our model was able to evaluate the nuances of each sentence. For example, a sentence expressing appreciation for help from a patient’s family members and friends yielded a high predicted PSI of 0.45. In contrast, a situation where patients believed that their condition was improving despite experiencing adverse drug reactions yielded a modest predicted PSI of 0.18. Similarly, a sentence describing considerable hardships from radiotherapy yielded the lowest PSI, which was −0.58.

**Table 1:** Predicted patient satisfaction index (PSI) corresponding to sentences in tōbyōki blogs.

Rank	PSI sentence examples^a^	PSI
1	Blindsided by the notification of my illness, I found immense joy and amazement in being able to accomplish so many things in just 6 months, with all of my friends waiting for me, bolstering my happiness.	0.45
2	Perhaps the new anti-nausea medication is working better than expected.	0.41
17	The results of the echocardiogram showed no abnormalities at all.	0.23
25	The only reason I can manage to tolerate the side effects is that I believe my condition is becoming better.	0.18
105	I told the doctor I wanted a chest scan, but he said he would not do it until I had more symptoms.	−0.11
149	I really do not like tests, and no matter how many times I have them, I never get used to them.	−0.33
171	I have had a number of severe depressive episodes, and I have also suffered from menopausal symptoms caused by hormone therapy.	−0.52
172	The radiation treatment was quite painful.	−0.58

^a^These are translations of original sentences written in Japanese. Some minor changes have been made to protect patient privacy. Ranks represent the results ordered from the highest to the lowest predicted PSI for the test set.

### Changes in PSI After Cancer Notification

**Figure 3:**
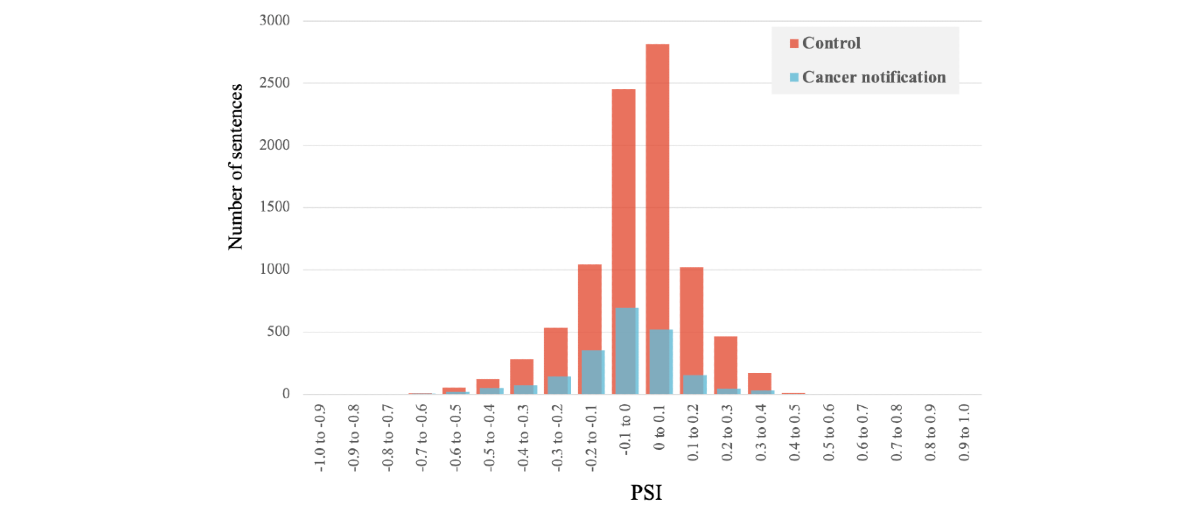
Distribution of the predicted patient satisfaction index (PSI) before and after cancer notification. Distribution of the predicted PSI at the time of and before cancer notification (defined as the control period). The figure shows the predicted PSI for each sentence in each period.

To confirm the applicability of this study’s BERT model in assessing the PSI for actual cases, we compared the PSI distribution between the time of cancer notification and the time before cancer notification using 48 *tōbyōki* blogs written by patients with breast cancer. Many sentences tended to have neutral PSIs (the proportions of PSIs between −0.2 and 0.2 were 1725/2097, 82.3% in the cancer notification period and 7330/8983, 81.6% in the control period; Figure 3). The proportion of sentences showing a negative PSI was higher in the cancer notification period (1342/2097, 64%) than in the control period (4497/8983, 50.1%). In addition, the negative PSI distribution in each sentence was slightly higher in the cancer notification period (mode –0.05) than in the control period (mode 0.05). These results indicate that many sentences in *tōbyōki* blogs had a neutral tone, even when they were written about cancer notification. Overall, the PSI was lower in the cancer notification period than in the control period.

**Figure 4:**
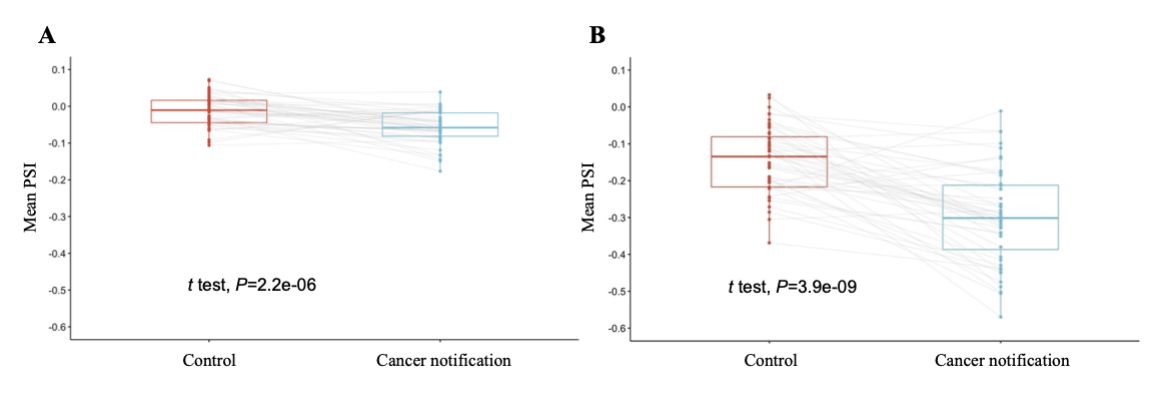
Patient satisfaction index (PSI) comparison before and after cancer notification. (A) Comparison of the mean PSI in the cancer notification and control periods without limiting. (B) Comparison of the mean PSI in the cancer notification and control periods limited to the 5 sentences with the lowest PSI values.

To examine the aforementioned differences in detail, we compared the mean PSI values at each period. Although the SD of the PSI distribution was similar in the cancer notification (SD 0.042) and control (SD 0.045) periods, the mean PSI was significantly lower in the cancer notification period than in the control period (−0.057 and −0.012, respectively; *t*_47_==5.392; *P*<.001; Figure 4A). This result suggests that cancer notification adversely affects the PSI, as expected. However, the average negative effect associated with cancer notification may have been partly diminished owing to the many neutral sentences in the cancer notification and control periods, as shown in Figure 3. This suggests that some sentences expressing shock concerning the notification may be overlooked when relying on the overall mean PSI alone. Hence, we focused on the sentences with the lowest PSI values, which were expected to highlight the most negative effects associated with cancer notification. The mean difference in the PSI between the cancer notification and control periods became apparent when the bottom 5 sentences were compared (−0.298 and −0.144, respectively; *t*_47_=7.214; *P*<.001; Figure 4B). Furthermore, the SD of the PSI distribution was larger during the cancer notification period (SD 0.128) than during the control period (SD 0.090). These results suggest that focusing on the lower end of the PSI successfully highlights the negative effects of cancer notification.

## Discussion

### Principal Findings

This study showed the successful development of a model that quantifies patient satisfaction based on patient-written textual data. The most recent studies on patient satisfaction have used relatively small samples [34,35]. However, our approach, which involved the use of an ML model with a vast amount of patient-written text from the internet, presented a realistic method for estimating patient satisfaction that can help improve health care services.

When we applied the neural network-based BERT model, the predicted PSI was strongly correlated with that judged by human interpretation (ρ=0.832; Figure 2), and the model became more reliable with pretraining (ρ=0.554; Figure S4 in Multimedia Appendix 1). This indicates the importance of pretraining specific to patients’ experiences in PSI prediction; therefore, pretraining using a large amount of data is essential to achieve high accuracy. Although a recent systematic review showed that there are many studies on NLP and ML using patient-written free texts [36], few studies have attempted text-based quantification, and no previous studies are available that could provide a benchmark for comparison with our model. Nevertheless, in 1 study on hospital care that evaluated the correlation between scores obtained from a patient questionnaire and those derived from sentiment analysis of free text comments, Spearman correlation coefficient did not exceed 0.50 [37], indicating the difficulty in achieving high accuracy. These findings also support our model’s excellent effectiveness in such a situation. Moreover, when we reviewed the sentences paired with PSIs (Table 1), we found that the model’s predictions considered the degree of meaning (the nuances of the language expressions) in the sentences, which is difficult to predict using conventional sentiment analysis. Collectively, these findings suggest that this model, which was trained on an immensely high volume of data from *tōbyōki* blogs, can be practically applied to determine accurate PSIs.

**Figure 5:**
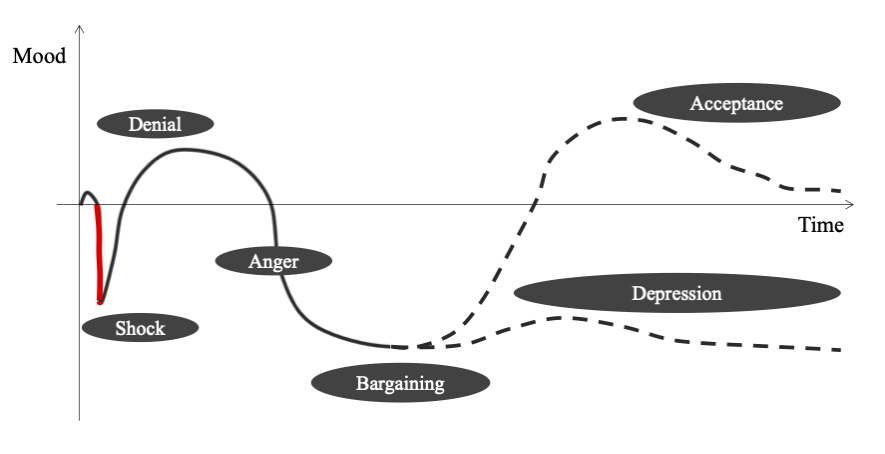
The 5-stage model of death and dying by Elisabeth Kübler-Ross. The thick red line denotes the “shock” induced by cancer notification. The dotted line indicates a fork in the path between acceptance and depression.

We validated the model’s applicability in assessing emotional responses to cancer notification, which usually occurs in the early phase of patients’ cancer journey. As expected, PSI was significantly lower during the cancer notification period than during the preceding control period (Figure 4), indicating that the model can quantify the psychological and emotional changes associated with the notification. When limited to the 5 lowest-ranking sentences, the difference in PSIs between the cancer notification and control periods increased, suggesting that the amplitude of negative emotions is high for some people, as reflected by the larger SD in the cancer notification period (SD 0.128) than in the control period (SD 0.090). Understanding patient satisfaction at each stage of treatment during routine clinical practice is essential for improving the medical care system in a patient-centered fashion because there may be discrepancies in the disease burden recognized by patients and clinicians [38]. Moreover, this result is consistent with Elisabeth Kübler-Ross’s 5-stage model of death and dying (Figure 5) [39]. In the Kübler-Ross model, a cancer notification induces a “shock,” represented as the steepest negative impact (shown as a thick red line). Although several factors, including both personal (eg, age, sex, and religion) and country (eg, country’s health policies and medical environment) levels, affect patient satisfaction, it is reasonable to assume that cancer notification is universally considered to be a negative event that causes a decline in patient satisfaction regardless of the differences in these factors. To our knowledge, our model succeeded in numerically expressing this phenomenon for the first time.

Our method of quantitatively identifying cancer-related anxieties can greatly contribute to future NLP research by highlighting patients’ medical needs efficiently. Although research using qualitative approaches, such as content analysis, is helpful in analyzing feedback from patients, qualitative review tends to be time-consuming and is associated with many challenges in analyzing information. Our approach, which is based on supervised ML using BERT, can significantly reduce the cost of human review. While many studies have reported achieving good results by applying BERT to both classification [40,41] and regression tasks [42], this study brings a unique perspective by demonstrating the potential usefulness of BERT in predicting patient satisfaction using a regression approach on patient-written textual data.

The current findings suggest that our model can be applied as a quantitative index of patient satisfaction not only in the field of PV but also in the field of health care services, where it can be used to determine the effectiveness of communication with health service providers and other related factors [43]. Considering that many hospitals in the United States continue to estimate patient satisfaction with treatment using questionnaires or Twitter [44,45], our approach represents a major breakthrough. Patients may be dissatisfied and anxious about services, including treatments, in their disease-fighting experience. In addition, this model can be applied to diverse areas of research subjects in social psychology, where quantitative consideration of population-level patient emotions may play an essential role in advancing knowledge in the field.

### Limitations

This study has several limitations. First, when applying our method cross-culturally, it is necessary to consider that the writers’ thoughts, culture, beliefs, and customs affect their content. Second, in our model, we exclusively used breast cancer data; therefore, the model may not be generalizable to other diseases, especially those that affect male patients. Therefore, as a scope for future work, data relating to both male and female patients should be analyzed using blogs on other diseases. Third, patient satisfaction inherently contains some level of subjectivity. It encompasses multiple facets, such as treatment outcomes, communication experience with health care providers, patients’ understanding of their disease, and the overall patient experience. Although this study focuses on an overall assessment of their disease condition and treatment reflected through their blog entries, our approach does not necessarily capture all aspects of patient satisfaction. Fourth, the subjectivity inherent in manual labeling procedures and the limitation of having a relatively small number of manual labels represent another constraint. Although we took careful measures to reduce potential bias and inconsistency, this aspect is subject to individual interpretation and understanding. Finally, we recognize the limitation in our definition of the “cancer notification period.” In our approach, 1 of our researchers manually read and verified the relevance of the content of the selected blogs to cancer notification. While this manual review ensured that the selected blogs were indeed about cancer notification, there may exist instances of cancer notification that use different terminology. Consequently, there may be missed cases where cancer notification is described using alternative expressions.

### Conclusions

We have proposed a distinctive approach for estimating patient satisfaction using patient-written textual data. Visualizing the patient’s journey and determining the causes of varying patient satisfaction will identify problems in routine medical practice and provide services to resolve these problems.

## Appendix

Multimedia Appendix 1 eTables and eFigures.

## Abbreviations

BERT: bidirectional encoder representation from transformers
ML: machine learning
NLP: natural language processing
PSI: patient satisfaction index
PV: pharmacovigilance
QOL: quality of life
RMSE: root-mean-squared error
SNS: social networking service
