# Supplementary material for: Estimating Patient Satisfaction Through a Language Processing Model: Model Development and Evaluation
Source: JMIR Form Res. 2023 Sep 14;7:e48534. doi: 10.2196/48534 (PMC10540017; doi:10.2196/48534)

Supplemental Material

**Estimating Patient Satisfaction through a Language Processing Model: Model Development and Evaluation**

Shinichi Matsuda*, Takumi Ohtomo, Masaru Okuyama, Hiraku Miyake, Kotonari Aoki

*Corresponding author.

Email: matsudasni@chugai-pharm.co.jp

**Table S1.** Pre-processing activities

**Table S2.** PSI annotation guideline

**Table S3.** Statistics for annotation results

**Table S4.** Characteristics of the data for fine-tuning

**Table S5.** The detailed procedure for computing PSI

**Figure S1.** Data flowchart

**Figure S2.** Selection of *tōbyōki* blogs to examine the change in PSI after cancer notification

**Figure S3.** Correlation between actual PSI and predicted score obtained by sentiment analysis

**Figure S4.** Correlation between the predicted and actual PSI using the Japanese BERT model without pre-training

**Table S1**. Pre-processing activities.

| Normalization of special symbols and space mismatches |
| --- |
| Removal of website URLs |
| Unification of alphabetic characters to lowercase |
| Unification of Japanese half-width characters to full-width characters using “mojimoji” in the python package (version 0.0.11) |
| An open-source Japanese morphologic analysis tool, Juman,^25^ was used to split sentences into morphemes. Morphological analysis is commonly used to delimit words in sentences comprising morphemes that are not separated by spaces, as is the case for Japanese writing. |
| Removal of some numerals satisfying either of the following conditions: numerals with less than 20 occurrences, numerals with more than 15 characters, and numerals with a score of less than 0.8 in the term-frequency inverse-document frequencies (tf-idf) score |

**Table S2**. PSI annotation guideline.

| Determine PSI in the range of -1.00 to +1.00. |
| --- |
| Score the experience described as positive if it is described favorably or negative if it is described negatively. |
| The PSI is determined by taking into account the nuances of the linguistic expression. As an example, the expression "I was very sincerely disappointed" would be considered more dissatisfying than "I was disappointed" and given a lower PSI. If a treatment includes both positive and negative aspects, such as "There was some pain, but I feel better after the treatment, so it seems to be working," the PSI will be judged based on the totality of the experience. |
| If the experience described is only a factual statement without any indication of whether it was favorable or unfavorable, the judgment shall be “neutral.” For descriptions based on quotations from medical sources (e.g., books, treatment guidelines), the judgment shall be “neutral,” as the description is factual only. |
| In a case where only a factual statement without any indication of whether it was favorable or unfavorable, if the experience described is one that is usually assumed to have a negative impact in many cases, the judgment shall be negative (e.g., pain, death). |
| Context should not be inferred. Judgment is made on each given sentence unit. |

PSI, patient satisfaction index

**Table S3**. Statistics for annotation results.

|  | **Annotator #1** | **Annotator #2** | **Annotator #3** |
| --- | --- | --- | --- |
| Maximum PSI | 0.90 | 0.64 | 1.00 |
| Average PSI | -0.032 | -0.035 | -0.026 |
| Median PSI | 0.00 | 0.00 | 0.00 |
| Minimum PSI | -1.00 | -0.84 | -1.00 |
| SD for PSI | 0.18 | 0.22 | 0.38 |

PSI, patient satisfaction index; SD, standard deviation

**Table S4.** Characteristics of the data for fine-tuning.

| **Characteristics of the data for fine-tuning** | **Result** |
| --- | --- |
| Total number of sentences | 792 |
| Total number of words | 19,271 |
| Unique number of words | 3,486 |
| Maximum number of words in each sentence | 114 |
| Average number of words in each sentence | 3 |
| Median number of words in each sentence | 24.3 |

**Table S5**. The detailed procedure for computing PSI.

|  | The detailed procedure |
| --- | --- |
| PSI for the cancer notification period | We identified the blog entry corresponding to the cancer diagnosis notification period in each patient’s blog. Each blog entry consists of multiple sentences, and the number of sentences can differ for each patient. First, the predicted PSI was calculated for each sentence. Second, we obtained the mean PSI at the entry-level by averaging those PSI in each blog entry. This entry-level mean PSI was used as the PSI in the cancer diagnosis notification period. |
| PSI for the control period | As described in the Materials and Methods section, the control period was defined as the time covered by 10 entries posted before the 120 days preceding the cancer notification period. Thus, we identified 10 blog entries corresponding to the control period in each patient. Similar to the above calculation for the cancer notification period, the predicted PSI was calculated for each sentence. Then, we obtained the mean PSI at the entry-level by averaging those PSI in each blog entry. Because we have 10 blog entries in the control period, we obtained the period-level mean PSI by averaging ten entry-level PSI. This period-level mean PSI is used as the PSI in the control period. |
| PSI using the bottom five sentences | As described in the Results section, some sentences expressing shock concerning the notification may be overlooked when relying on the overall mean PSI alone. Hence, we further focused on the bottom five sentences with the lowest PSI values. After calculating the predicted PSI for each sentence, we selected the bottom five sentences in each entry. After that, the PSI in each period was calculated in the same manner as described above. |

PSI, patient satisfaction index

**Figure S1**. Data flowchart.

**Figure S2**. Selection of *tōbyōki* blogs to examine the change in PSI after cancer notification.

“Cancer notification period” was defined as the time when any blog entry containing the word “cancer notification” was posted; each of these entries was manually confirmed. In contrast, the “control period” was defined as the time covered by 10 entries posted in the 120 days preceding the cancer notification period. Since it was thought that the negative effects of anxiety about symptoms or examinations might have affected the period just before cancer notification, a fixed period (120 days) was added to capture the psychologically stable state. For example, pattern C was excluded because there were data for only fewer than 120 days before cancer notification.

**Figure S3**. Correlation between actual PSI and predicted score obtained by sentiment analysis.

Predicted versus actual PSI using the test set. PSI, patient satisfaction index


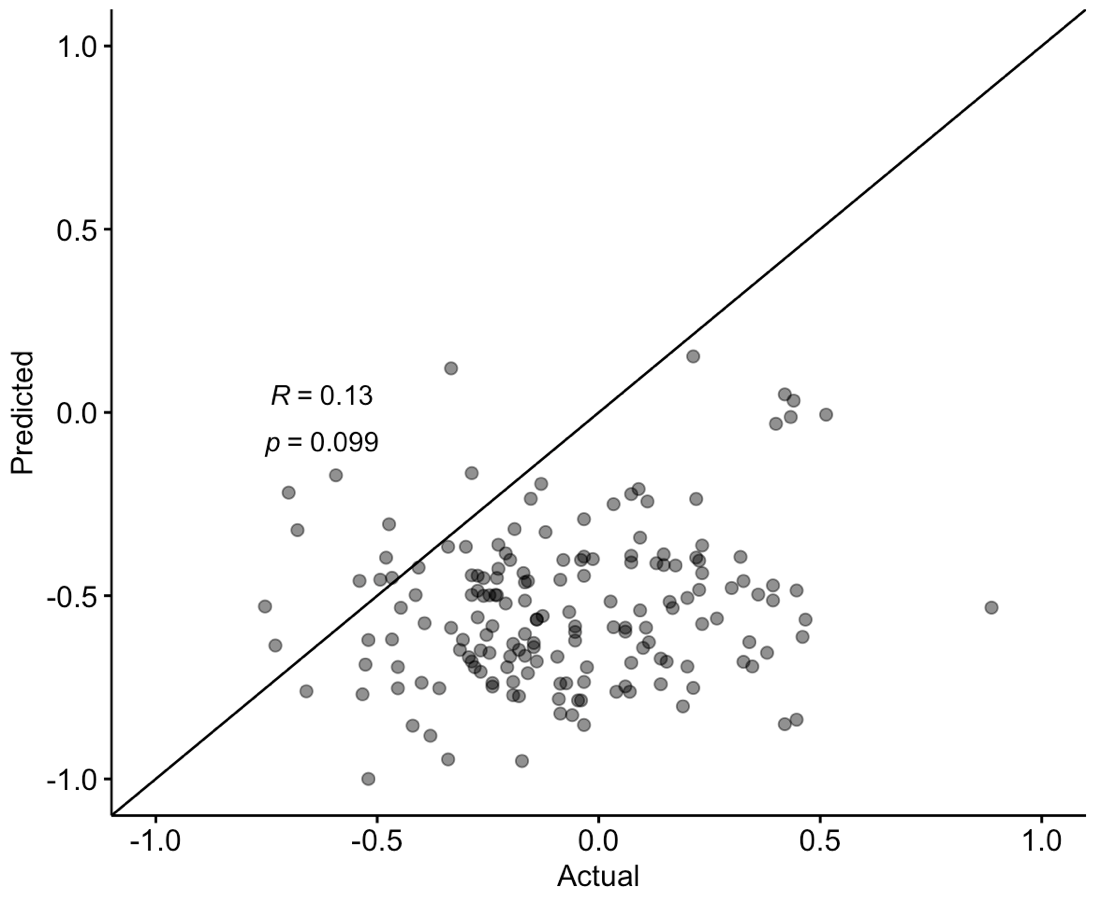


**Figure S4**. Correlation between the predicted and actual PSI using the Japanese BERT model without pre-training.

Predicted versus actual PSI using the test set. PSI, patient satisfaction index


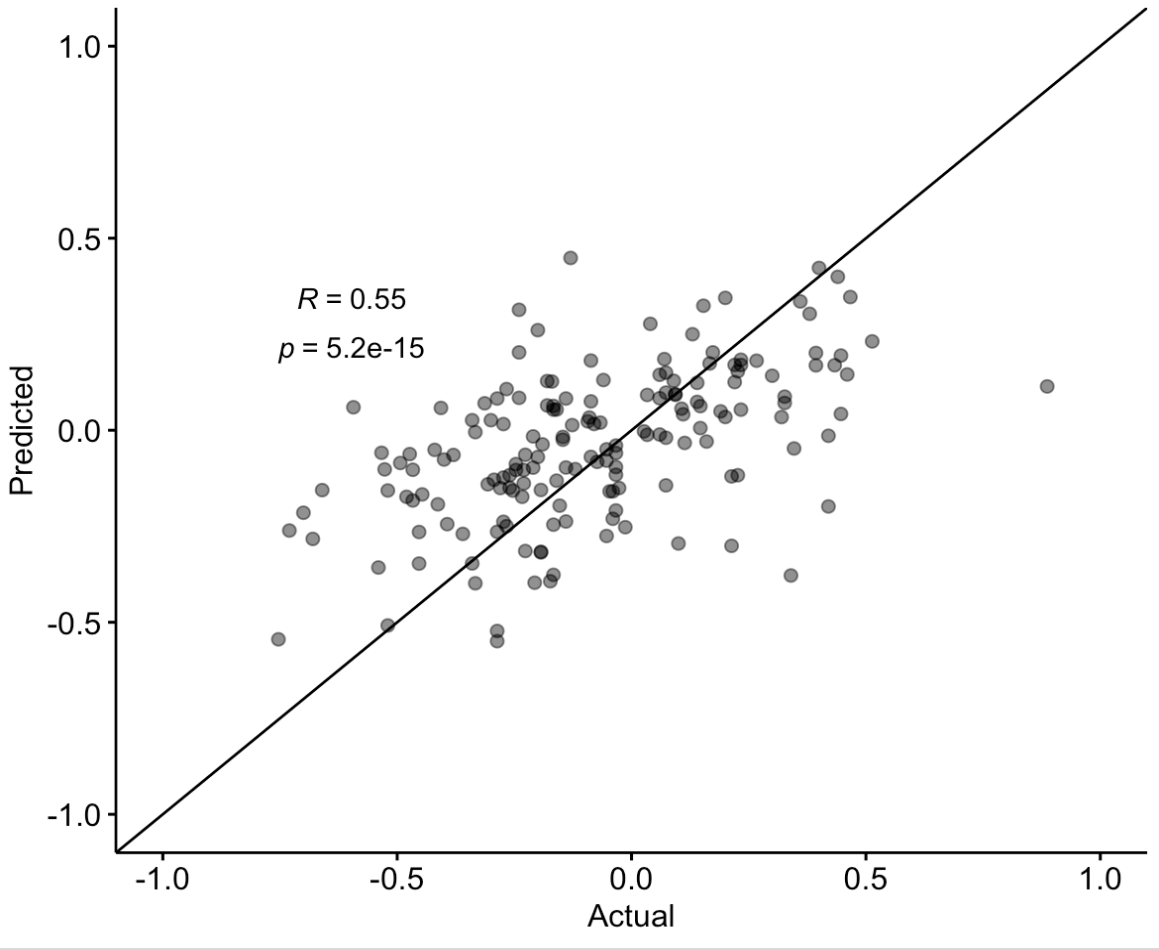

Supplement: Multimedia Appendix 1 [file formative_v7i1e48534_app1.docx]
